# Temperature Variability at Local Scale in the Bordeaux Area. Relations With Environmental Factors and Impact on Vine Phenology

**DOI:** 10.3389/fpls.2020.00515

**Published:** 2020-05-20

**Authors:** Laure de Rességuier, Séverine Mary, Renan Le Roux, Théo Petitjean, Hervé Quénol, Cornelis van Leeuwen

**Affiliations:** ^1^EGFV, Bordeaux Sciences Agro, INRAE, Université de Bordeaux, ISVV, Bordeaux, France; ^2^VITINNOV, Bordeaux Sciences Agro, ISVV, Bordeaux, France; ^3^CIRAD, Forêts et Sociétés, Montpellier, France; ^4^LETG-RENNES, UMR 6554 CNRS, Université Rennes 2, Rennes, France

**Keywords:** climate, local scale, viticulture, spatial modeling, vine development, phenology, terroir

## Abstract

Climate is a major factor of the physical environment influencing terroir expression in viticulture. Thermal conditions strongly impact vine development and grape composition. Spatializing this parameter at local scale allows for more refined vineyard management. In this study, temperature variability was investigated over an area of 19,233 ha within the appellations of Saint-Émilion, Pomerol, and their satellites (Bordeaux, France). A network of 90 temperature sensors was deployed inside grapevine canopies of this area and temperatures were measured from 2012 through 2018. To determine the effect of temperature on vine development, the phenological stages (budbreak, flowering, and véraison) were recorded on 60 reference plots planted with *Vitis vinifera* L. cv. Merlot located near the temperature sensors. Results showed great spatial variability in temperature, especially minimum temperature, with an amplitude of up to 10°C on a given day. The spatial variability of the Winkler index measured in the canopy inside a given vintage was around 320 degree-days. This research explores the main factors affecting spatial variability in temperature, such as environmental factors and meteorological conditions. The impact of temperature on vine behavior was also analyzed. Observed phenological dates were compared to those estimated using the Grapevine Flowering Véraison model. Maps of temperatures and phenological observations were created over this area and provided a useful tool for improved adaptation of plant material and training systems to local temperature variability and change.

## Introduction

Climate is a major factor of the physical environment influencing terroir expression in viticulture (van Leeuwen et al., 2004; Jones, 2018). Climate, and particularly temperature, determine to a large extent the growing areas well adapted for quality viticulture. Such areas are located mainly between the latitudes 30 and 50°N and 30 and 40°S, with average temperature ranging from 12 to 22°C across the growing season (Gladstones, 1992; Jones et al., 2012). Production of high quality wine grapes requires temperatures that allow ripening in a specific period of the year, ideally in September or early October in the northern hemisphere (van Leeuwen and Seguin, 2006). Extreme temperatures are not beneficial for vine development and grape quality. High temperatures (>35°C) can induce leaf or bunch damage, reduce photosynthesis, and decrease anthocyanin concentrations (Kriedemann and Smart, 1971; Spayd et al., 2002; Mori et al., 2007). Extreme negative temperatures (<-15°C) during winter are likely to cause permanent damage to wood and winter buds, possibly leading to vine death. Impact of negative winter temperatures depend on many parameters like genotype, environment, cultural practices, duration of frost exposure, and tissue hydration (Zabadal et al., 2007; Ferguson et al., 2014). Temperatures below −2.2°C after budbreak can damage young shoots and severely reduce production without, however, killing the vines (Poling, 2008; Dami et al., 2012).

Air temperature strongly impacts vine development and the timing of phenological stages (De Cortázar-Atauri et al., 2009; Parker et al., 2011, 2013, 2020; Chuine et al., 2013) and also grape composition (Mira and de Orduña, 2010). Sugar and acidity content at harvest are related to temperature (Coombe, 1987). This is also the case for secondary metabolites like anthocyanins, which increase with increased temperature up to a threshold and then decline (Spayd et al., 2002; Mori et al., 2007; Tarara et al., 2008). Temperature also impacts aromas and flavor precursors like metoxypyrazines (including IBMP; green pepper flavor), which decrease with higher temperature during the growing season (Falcão et al., 2007). Trimethyl dihydronapthalene (TDN; notes of kerosene), massoia lactone (dried figs and coconut flavors), and γ-nonalactone (cooked peaches flavor) concentrations, are higher in wines made from grapes ripened under warmer conditions (Marais et al., 1992; Pons et al., 2017) which is rather a negative effect on wine quality.

Considering that thermal conditions strongly impact vine development and grape composition, characterizing this parameter is highly important. Several temperature indicators were developed to characterize wine production areas. The Winkler and the Huglin indices (Winkler, 1974; Huglin and Schneider, 1998) or the Average Growing Season Temperature (Jones, 2006) are simple indicators based on the growing season air temperature and allow classification of wine producing areas. Depending on the objectives of climate zoning, it may be appropriate to use a multi-criteria approach (Tonietto and Carbonneau, 2004).

Climate varies temporally and spatially and the annual temporal variations impacting vine development and grape quality potential are considered part of the vintage effect (van Leeuwen et al., 2004; Ubalde et al., 2010). The spatial climate variability has an impact on grapevine variety distribution, vine training system, technical management, and wine styles (Gladstones, 2011). Climate can be reduced to several different scales from macroclimate to microclimate and these scales are inter-dependent (Hess, 1974; Neethling et al., 2019). The spatial variability of climate at a local scale can be highly important and in some cases even more so than variability at large scale, due the influence of local parameters like relief, human infrastructure, vegetation, or bodies of water (Quénol, 2014). The high local temperature variability also depends on the different energy transfer processes between the atmosphere and the surface, thus characterizing the energy balance. It is the ratio between energy input and losses that will determine the air temperature. The energy balance is strongly determined by surface characteristics and atmospheric conditions (solar radiation, cloud cover, wind conditions). The spatial variability of temperatures is higher in anticyclonic atmospheric situations (calm and clear skies) than in low pressure situations (cloudy skies and wind). Cloud cover and wind have a homogenizing effect on temperatures, which reduces the impact of surface characteristics (e.g., topography) on the spatial distribution of temperatures (Guyot, 1997). For these reasons, it is of interest to characterize climate at local scale in winegrowing areas.

The study of climate at local scales requires appropriate measurement networks and climate models. Climate has historically been studied at global and regional scales (continent, country, wide region) by using weather station data from national networks or simulated data from climate models. Weather stations only produce point data and the network mesh is not fine enough to study local climates. Over the past few years, many studies of applied climatology have required the installation of measurement networks at local scales and the development of modeling tools adapted to these scales (Joly et al., 2003; Stahl et al., 2006; Bonnefoy et al., 2013; Wu and Li, 2013; Quénol et al., 2017). Different types of models exist to represent climate at various scales. At the global scale, general circulation models (GCMs) are mainly used to build climate change scenarios that estimate trends in climate variables at low spatial resolution. Global climate models (GCMs) have a resolution of several tens to hundreds of kilometers (IPCC, 2013). Obviously, these types of models cannot take into account the influence of local effects related to surface characteristics. Downscaling methods are therefore used to integrate the effects of surface characteristics to increase the spatial resolution of the models (Daniels et al., 2012). Regional climate models (RCMs) are downscaled global climate models that aim to regionalize the outputs of global models by using nesting of model grids of increasing resolution (Rhoades et al., 2015). In viticulture research, regional climate models (RCMs) were used to produce climate maps at regional scales in Marlborough region in New Zealand (Sturman et al., 2017), in Stellenbosch winegrowing area in South Africa (Bonnardot and Cautenet, 2009), and in Burgundi (Xu et al., 2012). Recent technological development, including miniaturization of temperature sensors and shelters, development of weather stations, as well as the use of digital elevation models (DEMs), geographic information systems (GISs), geostatistics, linear, and non-linear regression modeling allow mapping of air temperatures across winegrowing areas at an even finer scale. Temperature variability was characterized at the regional scale by using weather station networks (Madelin and Beltrando, 2005; Bois, 2007; Cuccia, 2013). More recently, temperature variability was characterized at the local scale by using temperature sensor networks deployed in vineyards (Bonnardot et al., 2012; Bonnefoy, 2013; Le Roux et al., 2017a).

Precise knowledge of temperature distribution at high spatial resolution allows growers to optimize viticultural practices and selection of plant material according to the local conditions. This issue becomes even more strategic in a context of global warming, where growers need to adapt to spatial temperature variability and evolution over time. There is a wide consensus in the scientific community that the climate is changing (IPCC, 2013), and the recent increase of temperature has already affected vine development, advancing in particular the timing of phenological stages (Bock et al., 2011; Tomasi et al., 2011; Duchêne et al., 2012; van Leeuwen et al., 2019), and modifying grape composition resulting in higher levels of potential alcohol and reduced acidity (Duchêne and Schneider, 2005; van Leeuwen et al., 2019) and wine aromas (Pons et al., 2017).

Considering the evolution of climatic conditions and the objective to preserve quality potential and wine typicity, growers will have to adjust viticultural techniques such as leaf area to fruit weight ratio, timing of pruning, or modify rootstocks, cultivars, or clones (van Leeuwen and Destrac-Irvine, 2017).

In this context, temperature variability was investigated over an area of 19,233 ha within the appellations of Saint-Émilion, Pomerol and their satellites (Bordeaux, France) from 2012 through 2018. The objectives of this study are: (i) to analyze daily and seasonal spatial and temporal temperature variability at local scale, (ii) to create maps of temperature and agro-climatic indices through spatial modeling, (iii) to assess environmental factors impacting spatial temperature variability, (iv) to assess the impact of temperature on vine development and grape composition at ripeness by means of a phenological model, and (v) to create maps of the occurrence of phenological stages.

## Materials and Methods

### Study Area

**FIGURE 1:**
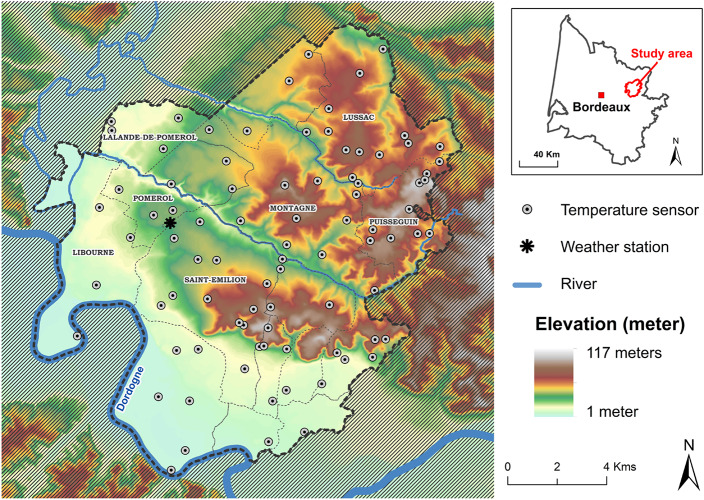
Location of temperature sensors and the weather station on a digital elevation model (IGN, National Geographical Institution, France).

Saint-Émilion, Pomerol, and their satellite appellations are located in the eastern part of the Bordeaux area, on the right bank of the Dordogne River, about 40 km of the town of Bordeaux (Figure 1). This area principally produces red wine and the main varieties grown are *Vitis vinifera* L. cv. Merlot (approximately 75%), *V. vinifera* L. cv. Cabernet franc (approximately 16%), and *V. vinifera* L. cv. Cabernet-Sauvignon (approximately 8%) (Cocks et al., 2014). Most vineyards are planted at densities between 5000 and 6000 vines per hectare. Vines are Guyot pruned and the training system is vertical shoot positioning (VSP trellis). For vineyard floor management, cover crop is widely used in particular on hillside vineyards to prevent erosion.

This area is characterized by several large tertiary (Paleogene) limestone plateaus at approximately 100 m in altitude, shaped by the erosion of the rivers which flow south (Dordogne) and north-west (Isle) of the study site. On the valley floors, gravelly and sandy soils have developed on quaternary alluvium (van Leeuwen et al., 1989). Relief was characterized by an altitude between 2 and 107 m, slopes up to 45% and various exposures.

**FIGURE 2:**
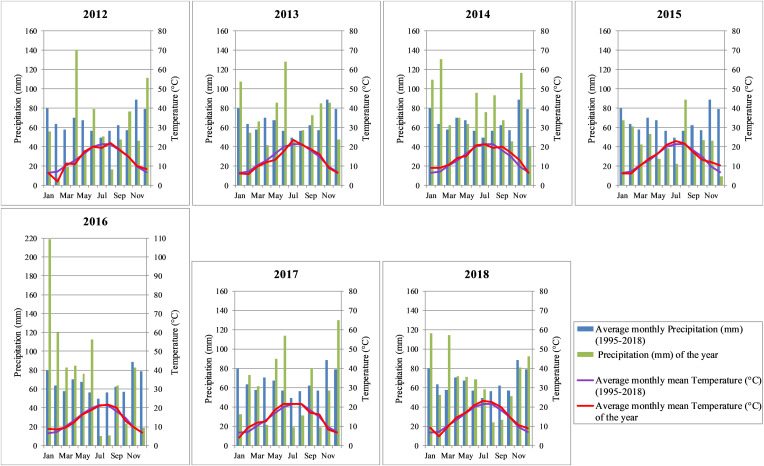
Annual distribution of temperature and rainfall from 2012 to 2018, in comparison to historical period (1995–2018), from the Saint-Émilion Météo-France weather station.

Climate is oceanic and temperate (Köppen and Geiger, 1954). Total yearly rainfall is 788mm ± 132.9, and the mean annual temperature is 13.9°C ± 0.5 (Data: Meteo France weather station of Saint-Émilion, average 1995–2018). Rainfall is well distributed all along the year but slightly lower in the summer (Figure 2).

### Temperature Monitoring

#### A Dense Network of Temperature Sensors

In order to characterize temperature variability over this area of 19,233 ha, including 12,200 ha of vineyards, a network of 90 temperature sensors was deployed at the end of 2011. This represents a density of one sensor for 214 ha. At this local scale, it is important to take into account the topography (exposure, slope, elevation), the latitude and longitude, and also local parameters, such as rivers and urban areas, which can potentially influence spatial distribution of temperature (Figure 1). Temperature sensors and data loggers were distributed to cover as much as possible the variability of these local parameters.

The data loggers used in this project from 2012 to 2015 are Tinytag Talk2-TK-4023 (Gemini Data Loggers, United Kingdom). From 2016 to 2018, automatic data recovery was achieved by using the LoRa technology, with new data loggers developed by OrbiWise company (Geneva, Switzerland). The thermistor probes used throughout the project from 2012 to 2018 were PB-5005-0M6 (Gemini Data Loggers, United Kingdom). The data loggers were set up on vine posts in the vineyard plots in order to measure temperature as close as possible to the vines. The probes were installed inside solar radiation shields (Type RS3), at a height of 1.2 m close to the vegetation. To reduce measurement errors due to vegetation located just around the solar radiation shields, vine shoots were regularly removed during the vegetative period (Madelin et al., 2014).

The data loggers recorded both minimum (Tn) and maximum (Tx) hourly temperatures. For data treatment, daily temperatures are used in this study. The daily minimum temperature corresponds to the extreme minimum temperature between the day before at 6 pm and the day at 7 pm, and the maximum temperature corresponds to the extreme maximum temperature between the day at 6 am and the day after at 7 am. Average daily temperature was computed as (*T*_*n*_ + *T*_*x*_)/2. Theoretical accuracy of the data logger is 0.4°C and to reduce measurement uncertainties, two thermistor probes were installed in every solar shield starting in 2017. This allows detecting deviation and erroneous temperatures due to sensor problems.

From 2012 to 2017, the quality of the minimum and maximum temperatures was graphically analyzed by plotting the daily data of all the sensors. Outliers and deviations were visually detected and eliminated of the data base. A good specific knowledge of field conditions was taken into consideration as not to delete extreme data recorded by specific sensors.

Since 2017, and due to the large number of data generated by the two probes installed in each solar radiation shield, a new methodology was used to streamline data processing. The absolute deviation from median was calculated for each sensor on each day. Then, the median absolute deviation (MAD) and its lower and upper bounds were calculated (Leys et al., 2013). MAD was multiplied by a constant (1.4826) linked to the assumption of normality of the data, to avoid the errors induced by outliers (Rousseeuw and Croux, 1993). Initial data were deleted if they were higher than the upper bound or lower than the lower bound calculated for each day. When no significant differences are observed, daily temperatures of both probes were averaged. This methodology was completed by plotting final data to visually detect remaining outliers and by controlling deleted data by comparing it to the initial data recorded by both probes.

For spatial modeling, outlier data were not recreated. For all other treatments, missing data were replaced, except when too much data was missing. If a temperature sensor has less than 30% missing data over the year and less than 20% over the growing season (April 1–September 30), then the data are recreated. For all days where the sensor produced values, deviation is calculated compared to the average of all other sensors of the network. This coefficient is subsequently applied to recreate daily missing data.

#### Weather Station

Meteorological data from the weather station of Météo-France, located in the Saint-Émilion area (Figure 1) were used to regionally characterize the studied years (2012–2018) (Figure 2). To compare the temperatures registered by this weather station and those recorded by the Tinytag data loggers located in vine parcels close to the canopy, a Tinytag data logger was set up in a vineyard plot at 4 meters distance to this weather station at the end of 2015.

#### Bioclimatic Indices

The Winkler degree day summation (Winkler, 1974) was used in this study, as it is well adapted to study the influence of temperature on vine development. This index is based on the sum of mean temperatures above 10°C, from 1 April to 31 October. Because temperatures were measured inside the canopy in this project, and not in a weather station as in Winkler (1974), this index is referred to a canopy Winkler index (CWI) (de Rességuier et al., 2018). CWI is based on the same formula as the Winkler index, the difference is due to the location where temperatures were recorded.

The GFV model (Parker et al., 2011, 2013) was created to simulate the occurrence of mid-flowering and mid-véraison from temperature data for a wide range of grape varieties. This model is based on a sum of daily mean temperatures above 0°C cumulated from the 60th day of the year (DOY). The date of mid-flowering and mid-véraison are determined when the thermal sum reaches a threshold value specific for each grapevine variety. The threshold values for Merlot are 1269 degree-days for mid-flowering and 2636 degree-days for mid-véraison, respectively (Parker et al., 2013). The dates that these threshold values were reached for flowering and véraison were calculated with data from the 90 temperature sensors. Results were compared to real phenology observations from 2012 through 2018 (excluding 2017 when a spring frost event severely damaged vine vegetation over the area).

### Vine Development Monitoring

To determine the impact of spatial temperature variability on vine development, the major phenological stages (mid-budbreak, mid-flowering, and mid-véraison) were measured for Merlot from 2013 through 2018 on blocks of 20 vines each at 60 locations near temperature sensors. Only 17 such blocks were surveyed during the 2012 vintage when the project was under development. The specific day when 50 percent of vine organs reached stage “C” for budbreak, stage “I” for flowering and stage “M” for véraison was recorded (Baggiolini, 1952; Destrac-Irvine et al., 2019). The 2017 vintage was excluded from the data analysis, because of frost damage which affected 80% of the reference plots.

Grape maturity dynamics were monitored on 18 blocks chosen to be representative of the thermal variability identified inside the study site. Every week, starting at véraison, grape berries were sampled and major grape metabolites were measured (Destrac et al., 2015). Maturity, which is highly dependent on winegrower decisions and intended wine style, is a phenological stage not easy to assess. Hence, the day when sugar content in grape berries reached 200 g/L was used as a proxy to characterize a theoretical maturity in order to compare the plots.

### Environmental Co-variables

Spatial temperature variability at the local scale is influenced by topography-related parameters (Carrega, 1994; Beltrando, 2000; Scherrer and Körner, 2011; Quénol and Bonnardot, 2014). A digital elevation model (DEM) provided by National Geographical Institution (IGN, France), with a 25 m horizontal resolution and 1 m vertical resolution, was used to produce raster layers of elevation, slope, and exposure by using the Spatial Analyst Tools from ArcGis software (ESRI, 92195 Meudon, France). Exposure was separated into two components (north/south and east/west) (Bonnefoy, 2013). Given the size of the study area (17 km^∗^18 km), the latitude and the longitude were also calculated from ArcGis software and taken into account as variables. Pixel values of all these environmental variables were extracted at the location of each temperature sensor.

### Statistical Analyses

The effect of environmental parameters (elevation, slope, north/south exposure, east/west exposure, latitude, longitude) on minimum temperatures on 13 March 2012 and 7 April 2012 were investigated by multiple linear regressions.

Average daily mean, minimum, and maximum temperatures over the growing season, CWI, and phenological observations were represented annually by using boxplot graph set up with the package ggplot2 from R software. Daily thermal amplitude on minimum and maximum temperature (years taken together) were represented monthly by using boxplot graph. In the boxplot representation, outliers are represented as dots. They correspond to observations whose values are higher than the value of the third quartile plus 1.5 times the interquartile interval, or less than the value of the first quartile minus 1.5 times the interquartile range.

Average daily mean, minimum, and maximum temperatures over the growing season and CWI were compared per year using a one-way ANOVA. Daily thermal amplitude on minimum and maximum temperature were compared per month using a one-way ANOVA. The data normality was checked using Kolmogorov–Smirnov one-sample tests and homoscedasticity using Bartlett’s test. When a significant effect of year on temperature was found, multiple comparisons were conducted to test differences between each year using Tukey’s HSD test (R package agricolae).

The effect of environmental parameters on minimum and maximum average daily temperatures and on CWI were analyzed by using linear mixed models (Pinheiro and Bates, 2000), where elevation, slope, north/south exposure, east/west exposure, latitude, and longitude are considered as fixed effects. Year was considered as a random effect accounting for replicates. Two-way interactions between elevation, slope, north/south exposure, and east/west exposure were measured. All analyses were carried out in R version 3.3.1 (R Core Team, 2016) using packages nlme and car.

Daily maps of minimum and maximum temperatures were created using support vector regression model (Le Roux et al., 2017a). Support vector regression is achieved by machine learning (Cortes and Vapnik, 1995) to estimate complex relationship between dependent variables and a series of predictors. The principle is based on automatic identification of a number of N support vectors (issued from data) between which the non-linear regression function will be estimated through a kernel K (here we used the Gaussian kernel). More complete details of the model and map production are provided in Le Roux et al. (2017a).

From pixels of subsequent daily maps created by the model, estimated dates of mid-flowering and mid-veraison were obtained by implementing the GFV model. A map of these phenological stage dates was produced each year and subsequently averaged over study period.

The root-mean-square error (RMSE) was used to compare mid-flowering and mid-véraison observations from each year (2012–18) with the mid-flowering and mid-véraison dates modeled using the GFV model and from maps produced from modeled mid-flowering and mid-véraison dates.

## Results

### Temperature Variability (2012–2018)

#### Daily Temperature Amplitude Analysis

**FIGURE 3:**
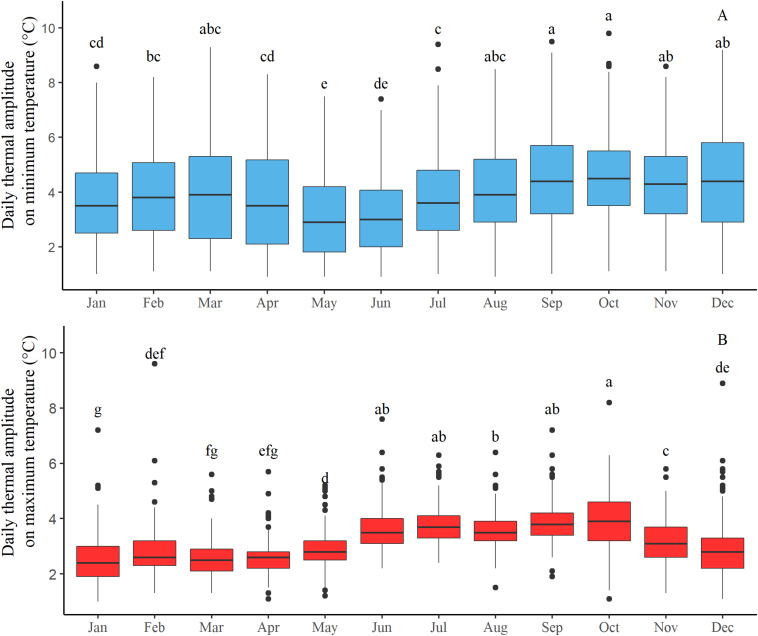
Monthly boxplots of the daily thermal amplitude for **(A)** minimum and **(B)** maximum temperature over the site from 2012 through 2018.

The spatial daily amplitude of minimum and maximum temperature (i.e., the difference between the highest and lowest minimum and highest and lowest maximum temperature) over the study area was analyzed from 2012 through 2018. Figure 3 represents the daily amplitudes per month (average of all years).

Spatial amplitude of minimum temperatures (average of 3.9°C ± 1.7, Figure 3A) is greater than for maximum temperature (average of 3.2°C ± 0.9, Figure 3B). The amplitude of minimum temperatures can reach 9.8°C on specific days, and the large size of the boxplot reveals important variations of amplitude from day to day (from 1 to 10°C).

Boxplot sizes of daily amplitude of maximum temperature are smaller, which indicated less variation from day to day, except some specific days (outlier points of the graph) (Figure 3B). Daily amplitude of maximum temperature presents also a seasonal effect, with greatest amplitude from June to October (a and b ANOVA groups).

**FIGURE 4:**
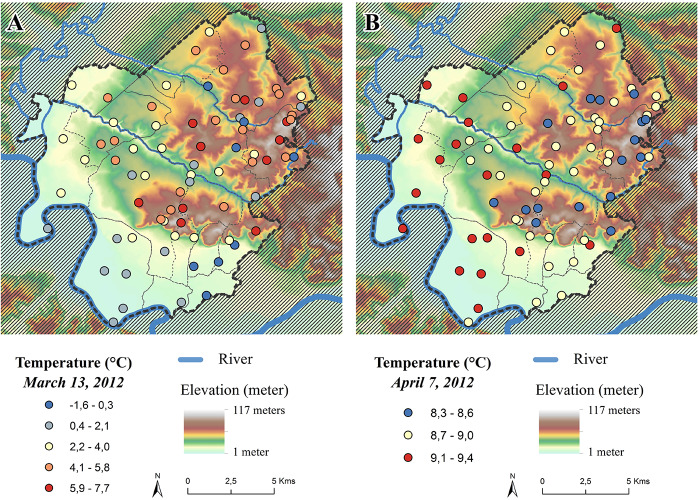
Maps of minimum temperature of 13 March **(A)** and 7 April **(B)** 2012.

The spatial structure of temperatures can be very different from day to day due to the weather, the atmospheric circulation, and the effects of the morphological features and land properties. In general, the spatial variability of temperatures is greater in atmospheric situations with clear skies and light winds. During clear sky days, more solar radiation reaches the surfaces and provokes higher levels of heating. During the night, clear sky induces stronger cooling due to greater long wave radiation emitted from surfaces. The distribution of minimum temperatures on 13 March 2012 was chosen as an example to illustrate the distribution of temperatures during an anticyclonic day, with clear sky conditions and no wind (Figure 4A). This weather type leads to large amplitude of minimum temperatures, by promoting cold air to flow into valleys and plains accentuated by long wave radiation emitted from surface. A minimum temperature amplitude of 9.2°C was recorded on this day, and relief is well correlated to this spatial distribution with coolest temperatures in the lowest parts of the valleys and warmest temperatures at the top of the hills on the limestone plateaus. Statistical analysis using multiple linear regressions of the minimum temperature of all the sensors against environmental parameters found elevation to be the most significant. Elevation positively explained 51.7% of the variance and latitude negatively explained 4.2%, for a total of 59% of the variance overall explained by the model. Conversely, a very different spatial minimum temperature distribution was observed on 7 April 2012, which was a cloudy atmospheric depression day with no wind (Figure 4B). This weather type reduces spatial thermal amplitude, which was restricted to only 1.1°C over the area. However, relief plays an important role on the minimum temperature distribution. During this day, 66% of the variance is negatively explained by elevation, 2.7% and 2.5% of the variance is positively explained by the east/west exposure and by longitude, respectively, and 1.5% of the variance is negatively explained by north/south exposure, with a total of 76% of the overall variance explained by the multiple linear regression model.

The effects of topography and other local factors are also combined with larger scale factors related to synoptic conditions (Planchon et al., 2015). The atmospheric circulation strongly influenced by the Atlantic Ocean generates a longitudinal gradient with higher temperatures over the western part of the site (Le Roux, 2017).

#### Temperature Variability Over the Vegetative Season

**FIGURE 5:**
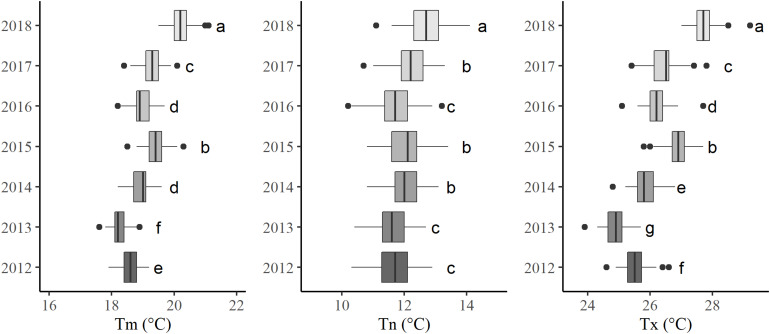
Boxplots of mean (Tm), minimum (Tn), and maximum (Tx) average daily temperatures over the growing season (from 1 April to 30 September) from 2012 through 2018. Different letters indicate significant differences between years (at *P* < 0.05).

Average daily mean, minimum, and maximum temperatures were analyzed during the growing season, from 1 April to 30 September during seven consecutive years (2012–2018) (Figure 5).

The average mean temperature is around 19.1°C ± 0.6 (with a mean amplitude of 1.5°C ± 0.2) and shows a marked vintage effect: 2018 is the warmest and 2013 the coolest year.

The intra-annual spatial variability, which corresponds to the range of temperatures between the coldest and the warmest sensor, is greater for minimum temperatures (2.6°C ± 0.3 in average over the seven vintages) than for maximum temperatures (2.1°C ± 0.3 in average).

The inter-annual (temporal) variability is greater for mean and maximum temperatures than for minimum temperatures. The multiple comparisons performed after the ANOVA found only three groups for the minimum temperatures in comparison to six and seven groups for the mean and maximum temperatures, respectively. Hence, at this site, the vintage effect is mainly driven by variations in maximum temperatures.

#### Canopy Winkler Index

**FIGURE 6:**
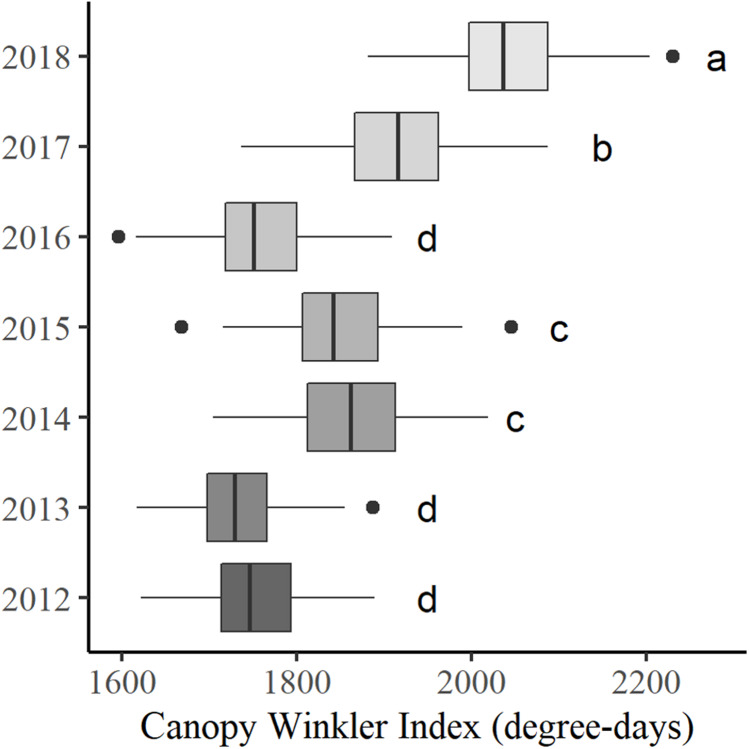
Boxplots of canopy Winkler index from 2012 through 2018. Different letters indicate significant differences between years (at *P* < 0.05).

In order to improve the characterization of temperature variability, the canopy Winkler degree-days summation was calculated for each sensor in each year (Figure 6).

An important vintage effect was highlighted with 2018 being the warmest and 2013 the coolest vintages, with CWI of 2041 degree-days ± 72.6 and 1735 degree-days ± 55.4, respectively. The spatial amplitude was also substantial, with an average of 320 degree-days ± 41.7 over the 7 years studied.

### Temperatures Modeling

#### Spatial Modeling of Daily Temperatures

**FIGURE 7:**
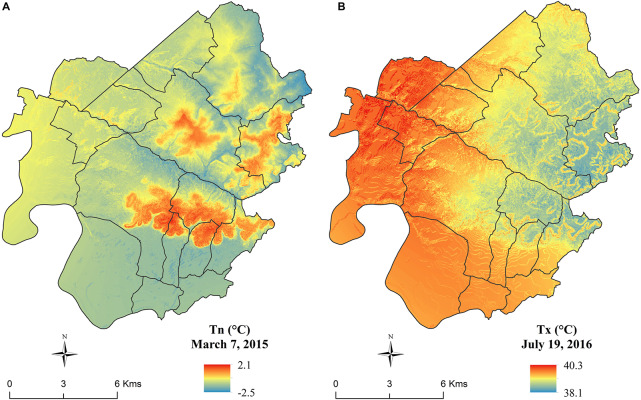
Spatial distribution of minimum temperature on 7 March 2015 **(A)** and of maximum temperature on 19 July 2016 **(B)**.

Minimum and maximum temperature maps were created for every day of the period under study (2012–2018). Figure 7 represents the spatial temperature distribution of 2 days with extreme conditions. 7 March 2015 was an anticyclonic day with clear weather without wind and rain, where temperatures dropped below 0°C in the areas most sensitive to cold air accumulation. 19 July 2016 was characterized by warm and dry weather, where the maximum temperatures of some areas located in the western and eastern parts of this area exceeded 40°C.

#### Spatial Distribution of Minimum and Maximum Temperatures During the Vegetative Season

Daily maps of minimum and maximum temperature were integrated over the growing season for each year. Independently of the vintage effect, a recurring spatial structure was shown (Le Roux et al., 2017b). It was therefore decided to average temperature maps over the duration of the study to quantify the temperature distribution for the purpose of producing a temperature zoning.

**FIGURE 8:**
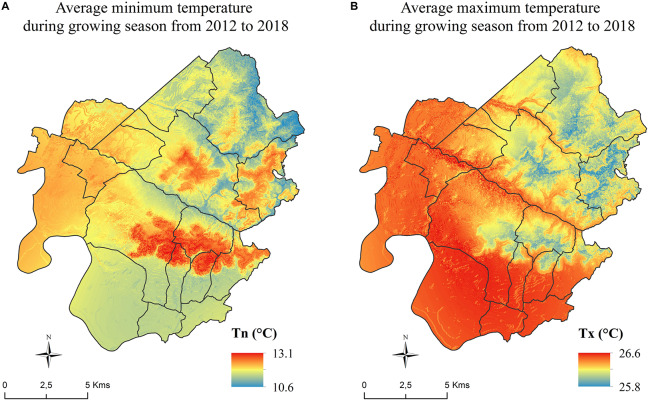
Spatial distribution of average **(A)** minimum and **(B)** maximum temperatures during the growing season (1 April to 30 September) from 2012 through 2018.

The analysis of the average minimum and maximum temperature maps over the study area shows a high spatial variability at this scale. For minimum temperatures, the sectors with the highest altitudes (limestone plateaus of Saint-Émilion, Montagne, Puisseguin, and Lussac), as well as those on south exposed slopes, correspond to the highest minimum temperatures (Figure 8A). Conversely, the lowest sectors (the Dordogne alluvial plain and the bottoms of the valleys) are associated with the lowest temperatures. The western part of the area (Libourne, Pomerol, Lalande-de-Pomerol) did not follow this distribution and minimum temperatures are slightly above average, while the altitudes are relatively low and the slopes close to zero.

For the spatial distribution of maximum temperatures, the opposite spatial pattern is observed: the warmest temperatures are recorded at low altitudes and coolest temperatures at high altitudes. High maximum temperatures are also recorded in the western part of the area (appellations Pomerol, Lalande de Pomerol, and the commune of Libourne, Figure 8B). The spatial thermal amplitude of the mean maximum temperatures is smaller compared to the minimum temperatures.

Finally, the areas with the greatest thermal amplitude between minimum and maximum temperatures are located at the bottom of the hills while the parcels located in the highest positions show smaller thermal amplitude.

#### Spatial Distribution of Canopy Winkler Index

**FIGURE 9:**
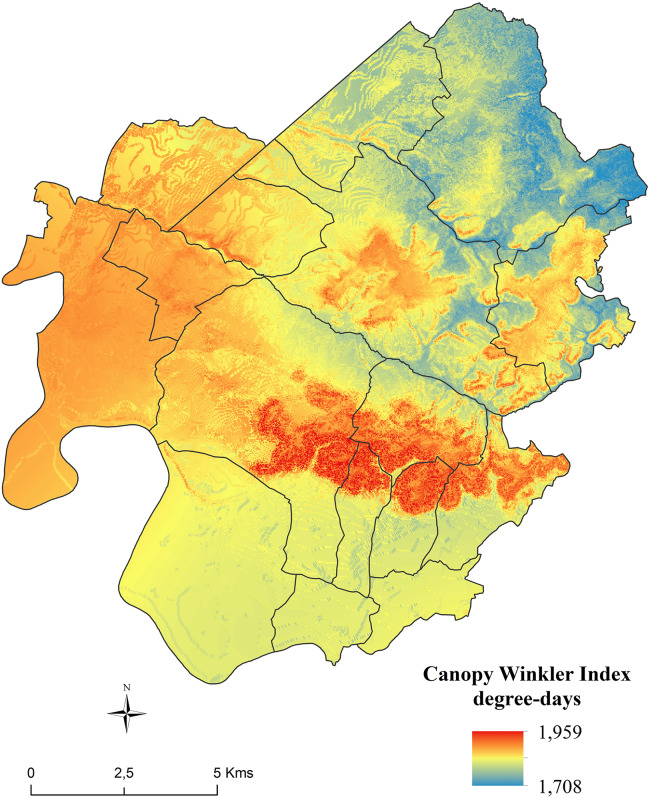
Spatial distribution of average Canopy Winkler Index (2012–2018).

The spatial distribution of the CWI (Figure 9) shows a spatial structure which is linked to the relief. The limestone plateau of Saint-Émilion and its south facing slopes are the warmest parts of the area. The north-east of the area is the coldest sector. The Dordogne alluvial plain and the bottom of the valleys are cooler. Another warm part of the region, not specifically linked to the topography, is the western part of the area around the town of Libourne, including Pomerol and Lalande dePomerol.

#### Environmental Factors Explaining Temperature Distribution

A statistical analysis was implemented to select the geomorphological co-variables which drive temperature distribution (Table 1). The main factors impacting minimum temperature over the vegetative season are elevation, longitude, slope, and latitude. Tn increased with elevation and the percentage of slope, and decreased from west to east and from south to north. Significant, but less important effects were found for exposure variables. The effect of elevation was, however, contingent on slope, and exposure parameters. For example, the negative coefficient parameter estimate for the interaction between elevation and slope indicated that the increase of minimum temperature with elevation is stronger in very steep vineyards than in parcels with low declivity.

**TABLE 1:** Summary of Linear Mixed-Models testing the effect of elevation, slope, exposure, latitude, and longitude on maximum temperature, minimum temperature, and Canopy Winkler index.

	Maximum temperature (°C)			Minimum temperature (°C)			Canopy Winkler index (degree-days)		
	Estimate	Std. error	χ^2^-value	Estimate	Std. error	χ^2^-value	Estimate	Std. error	χ^2^-value
Elevation (m)	−6.9E-03	9.0E-04	**126.0 (<0.001)**	2.3E-02	1.0E-03	**681.9 (<0.001)**	1.85E+00	1.37E-01	**174.6 (<0.001)**
Slope (%)	6.7E-02	1.6E-02	**16.6 (<0.001)**	1.1E-01	1.8E-02	**108.0 (<0.001)**	2.03E+01	2.38E+00	**133.0 (<0.001)**
South/north exposure	2.0E-01	5.1E-02	0.1 (0.7)	−2.5E-01	5.8E-02	**6.4 (0.01)**	−6.78E+00	3.07E+00	**4.9 (0.03)**
West/east exposure	−4.1E-01	6.8E-02	7.3 (0.007)	2.9E-01	7.6E-02	**14.8 (<0.001)**	3.28E+00	3.69E+00	0.8 (0.4)
Longitude (m)	−6.2E-07	5.0E-06	0.02 (0.9)	−1.3E-04	5.0E-06	**574.1 (<0.001)**	−1.00E-02	1.00E-03	**377.3 (<0.001)**
Latitude (m)	−1.0E-05	4.0E-06	1.9 (0.2)	−4.0E-05	5.0E-06	**68.0 (<0.001)**	−4.85E-03	1.00E-03	**62.7 (<0.001)**
Elevation (m) * slope (%)	−1.0E-03	3.2E-04	**10.3 (0.001)**	−1.2E-03	3.6E-04	**10.3 (0.001)**	−2.60E-01	4.90E-02	**28.8 (<0.001)**
Elevation (m) * south/north exposure	−3.5E-03	8.4E-04	**17.2 (<0.001)**	3.5E-03	9.5E-04	**13.2 (<0.001)**	/	/	/
Elevation (m) * west/east exposure	7.0E-03	1.3E-03	**29.7 (<0.001)**	−3.7E-03	1.4E-03	**6.6 (0.01)**	/	/	/

*P-values are indicated within brackets and significant effects are shown in bold.*

Regarding maximum temperatures, the main effect is elevation. Tx decreased with elevation and increased with percentage of slope. The effect of elevation was, however, contingent on slope, on south/north exposure, and on west/east exposure. Regarding the interaction between elevation and west/east exposure, the positive coefficient suggested that the effect of elevation on maximum temperatures was lower in east facing parcels.

Canopy Winkler index increased with elevation, slope, and decreased with north/south exposure, longitude, and latitude. The effect of elevation was concomitant with slope. The negative coefficient parameter for the interaction showed that the increase of CWI with elevation was higher in steep vineyards than for parcels with low declivity.

### Relationship Between Vine Development and Temperature

#### Phenological Observations

**FIGURE 10:**
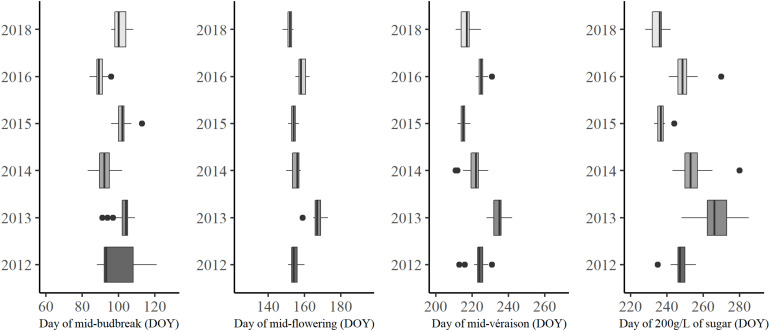
Boxplots of observed phenological stages from 2012 through 2018.

Phenology monitoring revealed the importance of the vintage effect: the warmer meteorological conditions of 2018 and 2015 advanced the timing of phenological stages compared to the cool year 2013 (Figure 10). The duration of phenophases between the phenological stages was variable from year to year. Some vintages, like 2016, can have an early budbreak due to high temperatures during the beginning of the year followed by a late flowering and véraison, because of relatively lower temperatures later in the growing season (Figure 2).

The intra-annual variability was highlighted in this study. An average window of about 19 days ± 7.7 was recorded for budbreak, 9 days ± 2.9 for flowering, 13 days ± 4.5 for véraison, and 25 days ± 11.3 for theoretical maturity (200 g/L of sugar content). The standard deviation showed more variation between years for budbreak and maturity, which is due to meteorological conditions affecting duration during these phenophases. For example, the maturity of the 2013 vintage was impacted by the poor ripening conditions that led to a delay in maturity. The 2012 budbreak was also affected by the cool temperatures at the beginning of the year and the rainy weather in April, which increased the duration of this stage.

#### The Timing of Mid-Flowering and Mid-Véraison Modeled by Means of the GFV Model

The GFV model was developed to predict the timing of flowering and véraison for a wide range of grapevine varieties. One of the objectives of this study was to produce occurrence maps of mid-flowering and mid-véraison dates by using the GFV model. To do so, the prediction accuracy of the GFV model for Merlot at this local scale needed to be validated first.

##### Temperature data correction

The GVF model was developed with data collected at regular weather stations. Data recorded by the Tinytag thermistor probe installed inside the vegetation were matched with the temperatures of the Météo-France weather station in order to compare the recorded temperatures.

**FIGURE 11:**
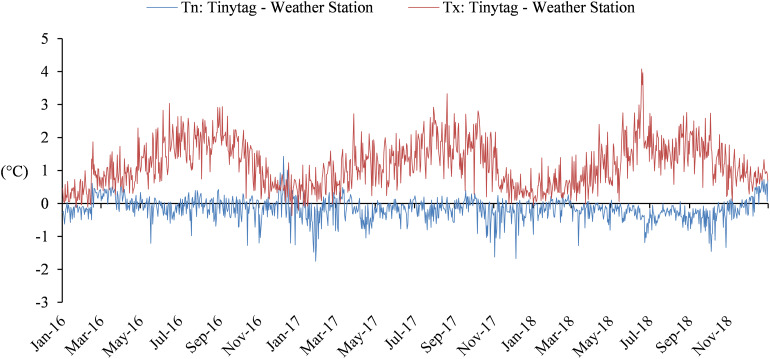
Comparison between temperatures recorded by Tinytag thermistor probe and the weather station of Saint-Émilion on daily minimum and maximum temperatures from 2016 through 2018.

Temperatures registered by both systems are different for minimum and maximum temperatures (Figure 11). The gap on minimum temperatures (average of −0.2°C ± 0.3) is less important compared to maximum temperatures (average of 1.2°C ± 0.7). A seasonal effect was also observed for maximum temperatures with greater differences during the vegetative season.

**FIGURE 12:**
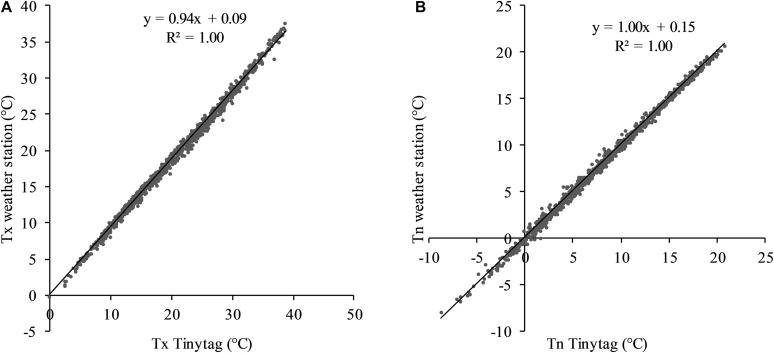
Relationships between minimum temperatures measured by Tinytag thermistor probe and weather station **(A)** and relationship between maximum temperatures measured by Tinytag thermistor probe and weather station **(B)**. Data cover 2016–2018.

Considering these differences, it appeared necessary to correct the data collected with the Tinytag data loggers in order to use the published GFV model parameters. A linear correction was shown to be satisfactory for minimum and maximum temperatures (Figures 12A,B).

The daily minimum and maximum temperature data recorded by Tinytag data loggers from 2012 to 2018 were corrected using these linear models in order to validate the GFV model.

##### Validation of GFV model

The GFV model was used to simulate the occurrence of mid-flowering and the mid-véraison for Merlot from corrected data of the 90 temperature sensors. Results were compared to the phenological observations to validate the performance of the GFV model at the scale of this site.

**FIGURE 13:**
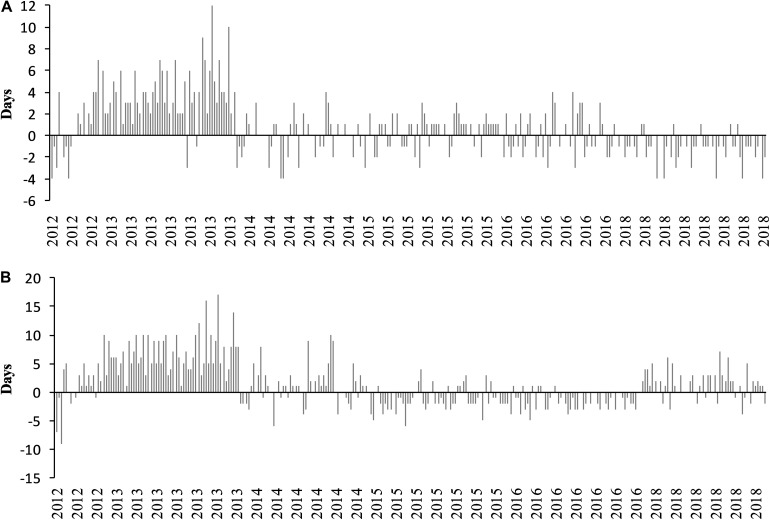
Differences between observed and predicted dates by using the GFV model for mid-flowering **(A)** and mid-véraison **(B)** from 2012 through 2018.

Figure 13 represents the differences between the observation and the GFV prediction for mid-flowering (A) and mid-véraison (B). The GFV model performed well at this local scale, with an accuracy of 4 days for mid-flowering including the 2013 vintage, which presents less precise results. Similar results were found for mid-véraison, the majority of the prediction errors are lower than 5 days, with an exception for the 2013 vintage. 2013 is a particular vintage with a very fresh and rainy beginning of the year (Figure 2). Flowering was affected by these adverse meteorological conditions, inducing poor fertilization which provoked coulure and millerandage and heterogeneous maturity. Storms in late July and early August provided unlimited water supply to the vines, causing continued vegetative growth and late and untypical ripening conditions.

Root-mean-square error was calculated for each year and phenological stage. Average RMSE (Table 2) is 2.2 days for flowering and 3.6 days for véraison, which shows that the GFV model is able to predict flowering and véraison with great accuracy at this scale. By comparison, RMSE of the GFV model for Merlot published in Parker et al. (2013) was 5.6 days for flowering and 6.6 days for véraison.

**TABLE 2:** RMSE calculated to compare mid-flowering and mid-véraison observations for each year (2012–18) with mid-flowering and mid-véraison dates modeled using corrected temperature data and from maps produced using modeled mid-flowering and mid-véraison dates.

Years	Number of flowering observations	RMSE (days) of flowering observations/flowering modeled from corrected temperatures by GFV	RMSE (days) of flowering observations/flowering dates extracted from a map created with modeled flowering dates using GFV	Number of véraison observations	RMSE (days) of véraison observations/véraison modeled from corrected temperatures by GFV	RMSE (days) of véraison observations/véraison dates extracted from a map created with modeled véraison dates using GFV
2012	17	2.2	2.3	17	3.7	3.7
2013	64	4.7	3.6	63	7.6	6.2
2014	59	1.7	2.4	59	3.4	3.5
2015	60	1.4	1.5	60	2.4	2.6
2016	58	1.8	1.8	60	2.2	2.3
2018	62	1.7	2.4	55	2.6	2.4
Mean (2012–18)		2.2	2.3		3.6	3.5
Std (2012–18)		1.2	0.7		2.0	1.5

##### Spatial modeling of the timing of mid-flowering and mid-véraison (2012–2018)

**FIGURE 14:**
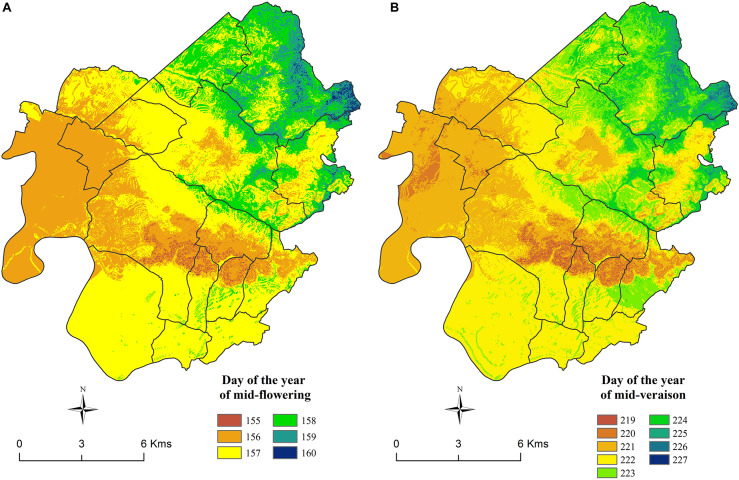
Maps of the average modeled occurrences of **(A)** mid-flowering and **(B)** mid-véraison stages (2012–2018) for Merlot.

Maps of spatial distribution of the occurrence of mid-flowering and mid-véraison stages averaged over the period 2012–2018 were created by using the daily maps of corrected temperature and the parameters of GFV model for Merlot (Figures 14A,B).

The spatial structure of the maps of both stages is similar and in adequacy with the spatial structure of CWI. The limestone plateaus, the south facing slopes, and the western part of the area have the earliest phenology overall. The northern and eastern part of the area, as well as the bottom of the valley (especially those located in the north eastern part of the area) show delayed phenology. The largest amplitude was found for the véraison stage, which is similar to the results from phenology monitoring (Figure 10). However, the spatial modeling reduces the amplitude across the area, compared to real observations from 9 to 6 days for mid-flowering dates and 13 to 9 days for mid-véraison dates.

##### Validation of phenological maps

To validate the phenology maps, an extraction of the pixel value at the location of each temperature sensor was carried out for each year, for flowering and véraison. Results were compared to phenology observations. The average RMSE is 2.3 days ± 0.7 for flowering and 3.5 days ± 1.5 for véraison, which corresponds to a similar result for observations of flowering and véraison compared to modeled flowering and véraison dates calculated directly using temperature data collected with the thermistor probes (Table 2). Hence, no accuracy loss was detected with the spatial modeling procedure.

## Discussion

### Spatio-Temporal Temperature Analysis

Based on a large unprecedented dataset obtained by a high density network of temperature observations, important spatial amplitude of seasonal temperatures over this area was observed. An average of 320 degree-days of amplitude over the 7 years studied was found for CWI.

This study also underlines large spatial amplitude on minimum temperatures during the vegetative season and great inter-annual variation of maximum temperatures, which impacts the vintage effect. Hence, spatial temperature variability was more driven by minimum temperatures, while temporal (i.e., year-to-year) temperature variability was more driven by maximum temperatures. Previous studies investigating temperatures at the vineyard scale have also shown great spatial temperature variability (Quénol et al., 2004; Bonnardot et al., 2012; Bonnefoy, 2013; Cuccia, 2013). These studies highlighted the impact of relief or local parameters such as water bodies on temperature distribution. This study shows not only the impact of relief, but also latitude and longitude. It confirms that great spatial thermal amplitude at local scale is well connected with local environment, as was also shown by Quénol et al. (2014) and Neethling et al. (2019).

Daily temperature analysis showed large spatial amplitude, especially on minimum temperatures. Spatial structure varies from day to day depending on the weather type, which confirms findings of other studies at the local scale (Madelin and Beltrando, 2005). The distribution of temperatures varies according to the atmospheric situation. Lower scale temperatures are also dependent on higher climatic scales. To explain the distribution of daily temperatures, it is necessary to study synoptic situations and their consequences on temperature distributions at the local scale. A preliminary study, based on data from this network over the period 2012–2015, showed an influence of weather types (Cantat et al., 2012) and atmospheric circulation patterns (Hess and Brezowsky, 1952) on the spatial and temporal daily variability (Eveno et al., 2016). This methodology was previously used is several studies to identify spatial climate variability (Douvinet et al., 2009; Planchon et al., 2009). Preliminary results in our study site show that great amplitude on minimum and maximum temperatures seems to result from northwest/north circulation, but also that warm weather induces great daily spatial amplitude on minimum temperature. These results need to be confirmed over the duration of the project with the use of appropriate statistical tools.

Another approach to assess the impact of weather conditions on spatial temperature variability is to classify daily temperature maps based on statistical criteria. In a second step, synoptic atmospheric conditions (wind direction and atmospheric pressure) of each cluster can be determined. This approach was tested on the 2014 data of this study site (Le Roux et al., 2017b). A classification in nine nodes of the spatial distribution of temperature was obtained. To determine the average daily atmospheric conditions of each node, the outputs of the regional model (Weather Research and Forecasting) were used. Spatial variability of temperatures was analyzed according to the atmospheric situation and allowed a better understanding of the results regarding the distribution of the temperatures over the study area. This approach was, however, carried out for only 1 year, and needs to be conducted over a longer period of time to confirm the observed relationships with increased statistical power.

In the future, it will be interesting to combine these two different approaches to better understand the influence of the weather type and the atmospheric situation on spatial temperature distribution at the local scale.

### Relationships Between Vine Development and Temperature

It is well established that temperature is a major driver of plant phenology (Chuine et al., 2013). Most studies assessing the relationship between temperatures and phenology are based on point data (i.e., data obtained in specific locations). Spatial modeling of phenological stages has been implemented, but only at large scale (Fraga et al., 2016; Sgubin et al., 2018). Our aim was to produce local scale maps of the occurrence of phenological stages. To do so, our spatial temperature modeling was coupled to phenology modeling. A high number of phenology observations were used as a validation dataset.

The GFV model (Parker et al., 2011) used to assess mid-flowering and mid-véraison dates was developed with data collected by regular weather stations. In this study, temperature data were recorded by Tinytag thermistor probes installed inside the vegetation. Hence, it was necessary to compare obtained data with temperatures recorded by a regular weather station. Temperatures registered by both systems are different for minimum and maximum temperatures. This result underlines influences of the local environment (vine parcel, canopy), but also an impact of intrinsic characteristics of measurement equipment (type of sensor, solar shield) on recorded temperatures. In order to use the published GFV model parameters (Parker et al., 2013) on the data collected with the Tinytag data loggers, temperatures collected in this study were corrected by means of a linear regression.

Several hundred real phenology observations allowed validation of the GFV model at this scale (RMSE = 2.2 and 3.6 days for mid-flowering and mid-véraison, respectively). Model performances were poorer in 2013, when unfavorable meteorological conditions during flowering induced fertilization problems (RMSE = 4.7 and 7.6 days for mid-flowering and mid-véraison, respectively). By coupling GFV and temperature models, maps of flowering and véraison were created and validated over this study area. These maps are highly accurate, as shown by comparing modeled phenology dates extracted from the maps with phenology observations (RMSE = 2.3 and 3.5 days for mid-flowering and mid-véraison, respectively). To improve precision, it would be interesting to develop site-specific phenology models, based on phenological observations and temperature data obtained over this area.

The GFV model performs well in current meteorological conditions, but it may be less accurate under much warmer conditions, because it is not capped for extreme temperatures. In a context of climate warming, and considering that phenology is the first biological indicator of climate change (Menzel et al., 2006), it will be interesting to also test models like the one created by Wang and Engel (1998), which identifies an optimal temperature and a critical threshold temperature above which plant development is stopped. This type of model is certainly more accurate to project phenology evolution over this site in extreme scenarios of climate change (RPC 8.5, end on the century).

To evaluate the impact of climate change on vine development, several studies were carried out in different areas (Cuccia, 2013; De Cortázar-Atauri et al., 2017; Alikadic et al., 2019). Coupling temperature projections under various climate change scenarios (IPCC, 2013) with spatial phenology modeling developed in this study will allow the creation of maps projecting phenological stages over this site.

### Comparing Amplitude of Temperature and Phenology Observations With Amplitude of Temperature and Phenology on Maps Obtained by Spatial Modeling

In our dataset, amplitude (i.e., the difference recorded by the coldest and warmest sensors) is 2.6°C for Tn, 2.1°C for Tx and 320 degree-days for CWI (average 2012–2018). On the maps obtained by spatial modeling, this amplitude is very similar for Tn with 2.5°C but is reduced to 0.8°C for Tx and 251 degree-days for CWI (average 2012–2018). Similar reduction is shown for phenological stages (from 9 to 6 days for mid-flowering dates and 13 to 9 days for mid-véraison dates).

The spatial modeling of the minimum temperature over the vegetative season is very accurate and the temperature ranges and the amplitudes are very close to the recorded temperatures. On the other hand, there is a sharp reduction of modeled amplitudes of maximum temperature. Visualization of the measured maximum temperature distribution over the different growing seasons (Figure 5) shows that the amplitudes are often extended by extreme points, which is not the case for the minimum temperature distribution. Extreme points on maximum temperature are often located in valleys close to vegetation like trees or hedges. Regarding the extreme coldest maximum temperatures, they are often located in higher elevation areas where environmental parameters favor air circulation and consequently temperature reduction.

The few extreme points influencing the large measured amplitude of maximum vegetative season temperature over the study area represent only a small weight in the spatial modeling and explain this reduction of amplitude after spatial modeling. The maximum temperature can be influenced by specific local environments like vegetation or wind, which are not taken into account in the modeling because they are not well represented. At this scale of modeling, and taking into account latitude/longitude and the relief parameters as co-variables, extreme points are prevented from having an important impact on spatial modeling.

It can be assumed that this amplitude reduction on the modeled maximum temperatures induced subsequently the loss of amplitude on the modeled CWI, which is created from compilation of daily modeled maps of Tn and Tx.

The reduction of amplitude of modeled phenology, compared to observed phenology, can result from decreased modeled temperature amplitude. Specific plant responses induced by factors other than temperature, like plant water or mineral status, plant age, clone or root-stock can, however, also impacted measured amplitude. These biotic and abiotic factors are not taken into account by the phenology models used in this research.

### Relationships Between Day–Night Temperature Amplitude and Wine Quality

High temperature amplitude (i.e., differences between day and night) during grape ripening is often considered to be a wine quality enhancing factor. This idea is frequently developed in popular wine books, and thermal amplitude during grape ripening is sometimes included in climatic characterization of wine producing areas (among other references see Montes et al., 2012). It is thought to increase secondary metabolites (phenolic compounds and aromas) in grapes and wines, although scientific evidence on this topic is scarce and somewhat contradictory. In one of the earlier references, Kliewer and Torres (1972) found that high anthocyanin accumulation in grape berries was related to *low* thermal amplitude. In contradiction with this, Mori et al. (2007) showed that, for a given day temperature, anthocyanins in grapes were higher when night temperature was low (i.e., in the case of higher day–night thermal amplitude). Regarding other phenolic compounds, Kliewer and Torres (1972) did not find a strong temperature amplitude effect on flavonols and so did Cohen et al. (2012) on proanthocyanidins. Over our study site, we found low temperature amplitudes on the limestone plateaus, where some of the finest wines of the area are produced, and high temperature amplitude in the valleys, known for producing entry level wine quality. This observation does not support the idea that high temperature amplitude is associated to high wine quality. Similar results were found by Bois et al. (2018) who recorded low day–night temperature amplitude in the Médoc area close to the Gironde Estuary (Bordeaux, France), where some of the finest Bordeaux wines are produced. Hence, inside the Bordeaux production area, high day–night temperature amplitude does not seem to be associated to high wine quality. More research is, however, required on this topic. Thermal amplitude is related to elevation and the proximity of water bodies, and so is soil distribution. It is possible that, in the case of the Bordeaux area, the effect of soil type overrules a potential impact of thermal amplitude. It is also possible that, independently from thermal amplitude, lower maximum temperatures promote grape quality potential: high temperatures induce cooked fruit aromas which are not associated with premium wine quality (Allamy et al., 2017; Pons et al., 2017).

### Terroir Characterization at Local Scale for a Better Adaptation of Current and Future Technical Management Strategies

Terroir is a concept based on the observation that wine quality and typicity are impacted by the physical and biological environment (van Leeuwen and Seguin, 2006). Major factors of the terroir effect are climate and soil (van Leeuwen et al., 2004; Bodin and Morlat, 2006; Morlat and Bodin, 2006; Jones, 2018). Terroir zoning is important for winegrowers in order to optimize the potential of their terroir by adapting plant material (rootstock and variety), training system, vineyard floor management, and harvest decisions to local climate and soil conditions (Reynolds et al., 2007; Vaudour et al., 2015; van Leeuwen and de Rességuier, 2018). Detailed soil maps are available for many winegrowing regions, including this study area (van Leeuwen et al., 1989; Swinchatt et al., 2018).

A previous study over the Bordeaux area produced temperature maps at 50 m of resolution (Bois, 2007; Bois et al., 2018). The results presented in our study increase the resolution of temperature mapping in a specific area of the Bordeaux wine region. To obtain this high resolution (25 m), a non-linear spatial model was developed based on the temperature data recorded by a high density temperature sensor network scale (Le Roux et al., 2017a). Maps of the different temperature indicators and agro-climatic indices, as well as maps of phenological stages, were produced in this research over the duration of the project and are well adapted to be used by large estates or cooperative cellars. These maps, and knowledge of local parameters involved in spatial temperature distribution, will help wine growers to better adapt plant material and viticultural practices. It will also allow them to determine harvest dates with increased precision. At very local scale, however, landscape features such as hedges or trees can influence temperature distribution (Quénol et al., 2014). These parameters have not been taken into account in our models. Hence, the interpretation of the temperature maps in these particular environments needs to be done with caution.

Climate change is heavily impacting viticulture (Schultz, 2000; Fraga et al., 2012; Hannah et al., 2013) and growers need to adapt to changing climatic conditions in order to continue the production of quality wines at economically sustainable yields (van Leeuwen and Destrac-Irvine, 2017). These adaptations include plant material (Duchêne, 2016), training systems (van Leeuwen et al., 2019), and pest management (Bois et al., 2017). In this context of climate change, it is also critical to better understand current climate, in order to establish a baseline for work on future adaptation.

Extreme weather events, such as frost or heat waves, can impact plant development and cause damage on vine organs (including grape berries) and alter wine quality and typicity. Daily maps of temperature distribution produced in this study can be used to better understand the spatial distribution of these extreme weather events as was done previously for frost event in Champagne and in South Africa (Madelin and Beltrando, 2005; Bonnardot et al., 2012). These maps could be used to define the most sensitive parcels in order to implement adaptations, or to optimize the location of systems like wind turbines for spring frost protection.

In the future, it will be interesting to combine temperature projections under various climate change scenarios (IPCC, 2013) with temperature model developed at the local scale (Le Roux et al., 2017a) in order to improve the accurate of the projections and to help winegrowers to anticipate adaptation.

## Conclusion

In this study, temperature variability was investigated at a vineyard scale over an area of 19,233 ha within the appellations of Saint-Émilion, Pomerol, and their satellites (Bordeaux, France). Results show a great spatial and temporal variability in temperature. The main factors driving this spatial temperature distribution, including environmental features and meteorological conditions, are explored. Impact of temperature on vine phenology was investigated by means of the coupling of spatial temperature models with phenology models. Local-scale maps of temperature and corresponding occurrence of phenological stages were created over this area. These maps allow improved adaptation of plant material and training systems to local temperature variability over the area. It is also a useful tool for adaptation of plant material and viticultural practices in the context of climate change.

## Data Availability Statement

The datasets generated for this study are available on request to the corresponding author.

## Author Contributions

LR conducted the study, contributed to data acquisition and statistical analysis, and elaborated the manuscript. TP contributed to data acquisition and temperature database development. SM contributed to the statistical analyses and wrote a part of the manuscript dedicated to statistics. RL contributed to the spatial modeling of temperature indicators and phenological stages. HQ and CL coordinated the study and participated in writing of the manuscript.

## Conflict of Interest

The authors declare that the research was conducted in the absence of any commercial or financial relationships that could be construed as a potential conflict of interest.
